# Circular RNA SIPA1L1 regulates osteoblastic differentiation of stem cells from apical papilla via miR-204-5p/ALPL pathway

**DOI:** 10.1186/s13287-020-01970-7

**Published:** 2020-11-02

**Authors:** Yuzhi Li, Minxia Bian, Zhou Zhou, Xiao Wu, Xingyun Ge, Tong Xiao, Jinhua Yu

**Affiliations:** 1 Key Laboratory of Oral Diseases of Jiangsu Province and Stomatological Institute of Nanjing Medical University, 136 Hanzhong Road, Nanjing, 210029 Jiangsu China; 2 Endodontic Department, School of Stomatology, Nanjing Medical University, Nanjing, Jiangsu China

**Keywords:** circRNAs, miR-204-5p, ALPL, SCAPs, Differentiation

## Abstract

**Background:**

Osteogenesis is a complex biological process which requires the coordination of multiple molecular mechanisms. This research aimed to explore the biological role and underlying regulatory mechanism of circSIPA1L1 during the osteogenic differentiation of stem cells from apical papilla (SCAPs).

**Methods:**

EdU retention assay, flow cytometry assay, and CCK-8 assay were used to evaluate the proliferation capacity of SCAPs. Western blot assay, alkaline phosphatase (ALP), and alizarin red staining (ARS) were conducted to investigate the biological roles of circSIPA1L1 and miR-204-5p. Fluorescence in situ hybridization was applied for circSIPA1L1 localization. Dual-luciferase reporter assay was performed to prove the interaction of circSIPA1L1 and miR-204-5p.

**Results:**

CircSIPA1L1 had no significant effect on the proliferative capacity of SCAPs. CircSIPA1L1 promotes osteogenic differentiation of SCAPs by serving as a miRNA sponge for miR-204-5p. Either knockdown of circSIPA1L1 or overexpression of miR-204-5p significantly suppresses osteogenic differentiation of SCAPs.

**Conclusions:**

CircSIPA1L1 upregulates ALPL through targeting miR-204-5p and promotes the osteogenic differentiation of SCAPs.

## Background

Cell-based tissue regeneration gives the field of regenerative medicine a boost. Mesenchymal stem cells (MSCs) present their superiority in individual treatment of tissue engineering based on their self-renew and multi-differentiation ability. They can differentiate into a variety of cell types such as osteocytes, chondrocytes, and adipocytes [1, 2]. As promising mesenchymal stem cells, SCAPs are isolated from human immature impacted permanent teeth, their essential roles in the dental pulp and dentin formation, alveolar bone regeneration, and tooth root growth have been extensively studied [3]. Increasing evidences have demonstrated that SCAPs could exhibit advantages of proliferative capacity and differentiation ability such as adipogenic, osteogenic, and neurogenic [4, 5]. Besides, many previous studies have indicated the bone tissue formation and regeneration capacities of SCAPs [6–8].

Circular RNAs (circRNAs) are a class of naturally occurring non-coding RNAs, which are abundant in the eukaryotic transcriptome and involve in regulating physiological processes [2, 9, 10]. Compared with traditional linear RNAs, circRNA molecules have a typical closed-loop structure, which is resistant to digestion with RNA exonuclease. The circular structure maintains its stability and strengthens its miRNA/protein-binding capacity [11, 12]. As a new molecular biomarker, circRNAs have brought fresh expectations for the diagnosis, treatment, and prognosis of clinical diseases [13]. However, circRNAs were considered to be byproducts of abnormal splicing with little functionality when they were first discovered 25 years ago. With the evolution of RNA deep sequencing technology and bioinformatics analyses, more and more circRNAs have been discovered in eukaryotes, and they present tissue-specific expression patterns [14, 15]. There is increasing evidence that circRNAs are indispensable in neuronal function, cancer progression, cell proliferation, and differentiation [16–18]. Generally, circRNAs act as microRNA’ (miRNA) sponges to binding with miRNAs and modulating mRNAs’ function. This regulatory process also called competitive endogenous RNAs (ceRNAs) mechanism. Recently, certain kinds of circRNAs have been verified to be involved in many cell biological processes, including the regulation of cell osteogenic differentiation via the circRNA-miRNAs-mRNA axis [19, 20].

MiRNAs are non-coding, single-stranded RNAs which regulate gene expressions through a post-transcriptional manner, resulting in translation inhibition or mRNA degradation [21]. As a key cell fate regulator, miRNAs are involved in critically important physiological processes of stem cells, such as cell proliferation and differentiation [1, 22, 23]. MiR-204-5p acts as a tumor suppressor to regulate the growth and metastasis of breast cancer and has antitumor effects on melanoma cells [24, 25]. Besides, studies have shown that miR-204-5p has the function of adipogenic differentiation [26]. In terms of osteogenic differentiation, a study proved that LncRNA TUG1 sponge miR-204-5p in calcific aortic valve disease then upregulated post-transcriptional expression of Runx2, leading to the promotion of osteogenic differentiation [27]. Moreover, Chen et al. demonstrated that miR-204-5p may be a potential upstream regulator factor in aging osteoblasts as it is significantly upregulated in an osteoporotic fractured bone. On the other hand, multiple indispensable genes related to osteoblasts differentiation and bone mineralization have been revealed. Among these genes, ALPL (alkaline phosphatase) which is recognized as an early expression gene associated with osteogenesis has attracted our attention [28].. The alkaline phosphatase (ALP) protein it encodes has been widely used as a diagnostic indicator to assess the grade osteoporosis [29]. Researches have shown that ALPL inhibits the sensitivity of bone aging by specifically regulating the differentiation and senescence of MSCs [30].

Our previous RNA-sequencing data revealed that different expression profiles of circRNAs, mRNAs, and miRNAs undergoing osteoblast differentiation of SCAPs. The competitive circRNA–miRNA–mRNA networks predicted by miRanda, RNAhybrid, and Target Scan software containing potential targeting relationships were comprehensively integrated. Among them, we obtained a regulatory axis constructed by circSIPA1L1, miR-204-5p, and ALPL [31]. We detected the expression of circSIPA1L1 during osteoblast differentiation of SCAPs. The results showed that the level of circSIPA1L1 is gradually increasing, which indicated that circSIPA1L1 may exert important functions in the osteoblast differentiation of SCAP. Importantly, in the present study, circSIPA1L1 has been confirmed to promote osteogenesis of dental pulp stem cells [32]. However, the function of circSIPA1L1 in the osteogenic differentiation of SCAPs has not been definitively elucidated yet. In this research, we found that circSIPA1L1 had a negative correlation with miR-204-5p and could promote osteogenic differentiation of SCAPs by upregulating the miR-204-5p target gene ALPL.

## Materials and methods

### Tissue collection and cell culture

Impacted human third molars were acquired from younger patients (17–20 years old) who need a tooth extraction for orthodontic reasons after signed informed consent. This research was supported by the Ethical Committee of the Stomatological School of Nanjing Medical University. The tissue was carefully isolated and clipped from the apical area of the immature root then digested with a mixture of 4 mg/mL trypsin (Gibco, Life Technologies, MA, USA) and 3 mg/mL type I collagenase (Gibco). Subsequently, SCAPs were cultured and identified as we previously reported methods [33]. The 3–5 passages of SCAPs were used in subsequent experiments.

### Adipogenic differentiation

SCAPs were planted into 6 cm culture dishes and cultured with an adipogenic inducing solution (Cyagen Biosciences Inc.) when the cell density reached 60–70%. Cells were cultured at 37 °C in 5% CO_2_ incubator, and the adipogenic differentiation medium was replaced regularly. After adipogenic induction for 28 days, cells were fixed with 4% paraformaldehyde (PFA) for 30 min and Oil Red O staining was carried out at room temperature.

### Chondrogenic differentiation

SCAPs (4 × 10^5^ cells) were collected and cultured in 15 ml centrifuge tubes with chondrogenic induction medium (Cyagen Biosciences Inc.) in a 37 °C, 5% CO_2_ incubator, and loosen the cap of the tube slightly. After 3–5 days, cell pellets were transferred to 24-well plates to culture for 20 days. Cell pellets were stained with Alcian Blue after fixation and frozen sections (5-μm thickness).

### Plasmid construction and cell transfection

SCAPs was transfected with miR-204-5p mimics (mimics, 50 nM) and mimics negative control (NC, 50 nM), miR-204-5p inhibitor (inhibitor, 100 nM), and inhibitor negative control (iNC, 100 nM) at 30–50% confluence with ribo*FECT*™ CP kit (Ribobio, Guangzhou, China). Similarly, the knockdown of circSIPA1L1 and negative control groups (si-SIPA1L1, si-NC, 100 nM) were also transfected in the same reagent. However, the circSIPA1L1 overexpression (sh-SIPA1L1, sh-NC) transfection was performed using lipofectamine 2000 (Invitrogen, USA). SCAPs were cultured with osteogenic induced medium (OM). Moreover, the circSIPA1L1 and ALPL luciferase reporter plasmids were constructed by site-directed mutagenesis and transfected with lipofectamine 2000 under serum-free medium in 293 T cells for 4–6 h then exchanged basic culture medium.

### Reverse transcription polymerase chain reaction (RT-PCR) analysis

SCAPs were transfected with several oligonucleotides mentioned above separately in an osteogenic induction medium. Then, total RNA was collected from SCAPs in each group by using TRIzol reagent (Invitrogen). The mRNA gets converted to cDNA by reverse transcription with a Prime Script RT Master Mix kit (TaKaRa, Dalian, China). For the analysis of the levels of miRNA, extracted RNA was specifically reverse transcribed using the transcription kit (Ribobio, Guangzhou, China). *GAPDH* was used as a housekeeping gene for mRNAs and circRNAs and *U6* was chosen as an endogenous control for miRNAs, and the data were calculated by 2^–ΔΔCT^ method as previously reported [34]. The primer sequences of the evaluated genes are listed in Table 1.

**Table 1:** Sense and antisense primers for real-time reverse transcription polymerase chain reaction

Genes	Primers	Sequences (5′-3′)
*ALPL*	Forward	ACCTGAGTGCCAGAGTGA
	Reverse	CTTCCTCCTTGTTGGGTT
*RUNX2*	Forward	TCTTAGAACAAATTCTGCCCTTT
	Reverse	TGCTTTGGTCTTGAAATCACA
*OSX*	Forward	CCTCCTCAGCTCACCTTCTC
	Reverse	GTTGGGAGCCCAAATAGAAA
*circSIPA1L1*	Forward	AAACTGGATGAACAAGGGAGAA
	Reverse	TGCTTCACTTTAACAGAGGGCTT
*GAPDH*	Forward	GAAGGTGAAGGTCGGAGTC
	Reverse	GAGATGGTGATGGGATTTC

### Western blot analysis

Three days after transfection, SCAPs were harvested and lysed in RIPA buffer after washing twice by PBS. The equivalent amount of proteins was added to 10% SDS-PAGE and transferred to PVDF membranes after separation. Then, 5% milk was used to block these membranes. Subsequently, the PVDF membranes were incubated with primary antibody: ALP (ab95462, Abcam), RUNX2 (ab76956, Abcam), OSX (ab22552, Abcam), and GAPDH (AP0060, Bioworld), each was diluted at 1:1000. The membranes were incubated for 1 h with secondary antibodies after washing with TBST for 30 min. Western Blotting Imaging System was used to detected immunoreactive bands.

### Immunofluorescence staining

After transfection and induction for 3 days, SCAPs were fixed with 4% PAF in 10 mm^2^ glass coverslips. Cells were permeabilized with Triton X-100 solution (Beijing, China) for 12 min and then blocked with goat serum at 37 °C for 1.5 h. After incubation with ALP and RUNX2 primary antibodies, they were treated with a mixture of secondary antibody with fluorochrome and phalloidin for 1.5 h in a dark at room temperature. DAPI (Beyotime, China) was applied to stained nuclei. Subsequently, ALP and RUNX2 were observed under the fluorescence microscope (Leica, Germany). ImageJ software was used for quantification.

### Alkaline phosphatase (ALP) staining and activity analysis

The experiment was performed after osteogenic induction for 5 days of transfected SCAPs. ALP staining kit (Beyotime, China) is used to qualitatively detect the expression of ALP as described previously [35]. ALP activity was examined with an ALP activity assay kit (Jiancheng, Nanjing, China). The absorbance was detected in the microplate reader at 520 nm. ALP activity was normalized to the total protein level.

### Alizarin red staining (ARS) and cetylpyridinium chloride (CPC) analysis

Mineralized nodule formation of transfected SCAPs in osteogenic-induced medium was visualized by ARS staining after osteogenic induction for 14 days. Following a fixation with 4% PAF and a wash with distilled water, SCAPs were dyed with 0.1% ARS (Sigma-Aldrich) for 30 min. To quantitatively evaluate mineralization nodules, 10% CPC (Sigma-Aldrich) was used to dissolve the nodules and the absorbance was detected at 560 nm.

### Flow cytometry

Trypsin without EDTA (Beyotime, Haimen, China) was used to collect the transfected cells when they reached 80%. After fixation with 75% pre-cooled ethanol at 4 °C overnight, the cell cycle phases (G0/G1, S, and G2/M) were analyzed using FACS flow cytometer after gently washing twice in 0.01% phosphate-buffered saline (PBS). The proliferative index (PI = G2M + S) was used to analyze results. Flow cytometry also used to identify SCAPs through cell surface makers. The transfected SCAPs were collected by trypsin and washed with PBS. The samples with different primary antibodies (CD29, CD34, CD45, CD73, CD90) were incubated for 15 min under dark conditions. Stained cells were analyzed after being rinsed twice with PBS.

### Cell proliferation assay

After SCAPs were transfected with si-SIPA1L1 or sh-SIPA1L1 and the corresponding negative control, cells were resuspended and reseeded on 96 well plates (2 × 10^3^ cells/well). Equal amount α-MEM containing 10% cell counting kit (CCK)-8 reagent was added into per well at days 0, 1, 3, 5, 7, 9, respectively, and incubated for 2 h under the same environmental conditions. The absorbance was measured at 450 nm with a microplate reader. The experiment was repeated in triplicate.

Cell-Light™ EdU Apollo®567 In Vitro Imaging Kit (Ribobio) was used for EdU assay. The transfected SCAP was seeded in a 24-well plate. Cells were fixed after 5 h incubation with reagent A. Before the cells were treated with 1 × Apollo® reaction mixture for 30 min, they were decolorized with 0.5% Triton X-100. Then, Hoechst 33342 was used to the stained cell nucleus for 30 min. Following a wash with PBS, dyeing results were visualized using a fluorescence microscope. ImageJ software was used cell count to calculate the cell proliferation rate.

### Nucleocytoplasmic separation

The experiment was carried out using the PARIS™ Kit as the manufacturer’s instructions. 10^7^ fresh cells were trypsinized and centrifuged at low speed. PBS was used to rinsed the cell pellet and then was discarded. The cells were resuspended in 400 μl of Cell Fractionation Buffer and incubated for 10 min. All experiment was conducted on ice all the time. Then, these samples were centrifuged for 1 min at 500×*g* to obtain nuclear pellet and cytoplasmic supernatant. Afterwards, the nuclear pellet was lysed with 400 μl of pre-cold Cell Disruption Buffer after carefully sucking out cytoplasmic fraction. Next, equal quantities of 2 × Lysis/Binding Solution were added to nuclear and cytoplasmic samples respectively at room temp and immediately mix thoroughly by inverting the tube several times or pipetting gently. The sample mixture was centrifuge for 1 min to pass the filter after mixing gently with anhydrous ethanol. Subsequently, the Wash Solution 1 and Wash Solution 2/3 were applied to complete the washing steps according to the instructions. Finally, the RNA was dissolved with Elution Solution preheated to 95–100 °C. The Nuclear RNA and cytoplasmic RNA were collected for qRT-PCR analysis.

### RNA fluorescence in situ hybridization (FISH)

To detect circSIPA1L1 localization in cells, SCAPs were cultured on slides in 24-well plates. Then, cells were treated with pre-cold 0.5% Triton X-100 for 5 min at 4 °C after fixation with 4% paraformaldehyde. 200 uL prehybridization was added into each hole to block at 37 °C for 30 min. At the same time, the hybridization buffer was preheated at 37 °C. Then, the FISH probe mixture was added to the hybridization buffer. Subsequently, the prehybridization was discarded, and SCAPs were incubated in the hybridization buffer containing FISH probe overnight in dark at 37 °C according to the instructions. Under the condition of avoiding light, cells were sequentially washed with hybridization buffer at 42 °C as the manufacturer’s instructions. Then, DAPI was re-dyed for 10 min in dark. After washed three times with PBS, the excessive liquid was removed. The images were captured using the LSM 710 confocal microscope (Leica, Germany).

### Luciferase reporter assay

Before plasmid transfection, HEK-293 T cells were seeded into 24-well plates (1 × 10^5^ cell/well). MiR-204-5p and negative control were transfected into HEK-293 T cells respectively on the second day. After 24 h of incubation, 100 ng circSIPA1L1 or ALPL wild-type reporter plasmid and 20 ng Renilla luciferase (RL) reporter plasmid was co-transfected into HEK-293 T cells when the cell density came up to 80%. The mutation of circSIPA1L1 or ALPL reporter plasmid was used as a control. Dual-Luciferase Reporter Assay System (Promega, Madison, WI) was used to detect the luciferase activity after 48 h.

### Statistical analysis

Based on all experiments conducted independently at least three times, the date was expressed as mean ± SD and analyzed using SPSS software and GraphPad Prism 5. We analyzed the statistically significant differences using Student’s *t* test and one-way analysis of variance. Image-Pro Plus 5.0 software was employed to measure the grayscale analyses [36]. In figures, NS = not significant, *P < 0.05 or lower was considered statistically significant.

## Results

### Identification of SCAPs and detection of the expression of circSIPA1L1 and miR-204-5p in SCAPs during osteoblast differentiation

**Fig. 1:**
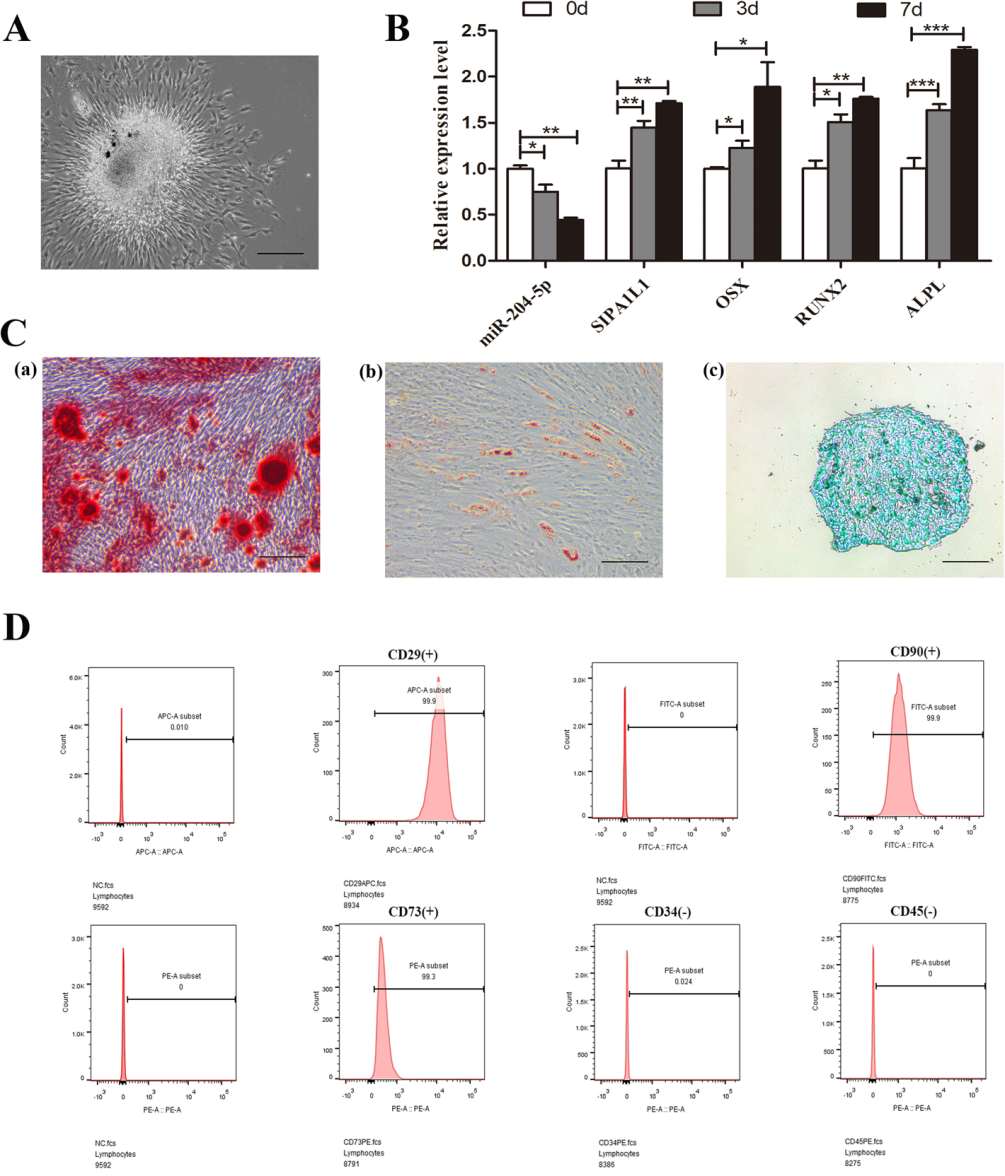
Identification of SCAPs and expression of circSIPA1L1 and miR-204-5p in SCAPs during osteoblast differentiation. **a** Morphology of primary SCAPs. **b** Relative gene expression of circSIPA1L1, miR-204-5p, and osteoblastic markers of *ALPL*, *RUNX2*, and *OSX* were detected by qRT-PCR at days 0, 3, and 7, respectively. **c** Tri-lineage differentiation of SCAPs was performed in vitro. **a** Alizarin red S staining of cells cultured for 14 days in osteogenic induction medium. **b** Oil red O staining of cells cultured for 28 days in adipogenic induction medium. **c** Alcian blue staining of cells cultured for 25 days in chondrogenic induction medium. (Scale bar = 200 μm.) **d** Flow cytometry demonstrated that SCAPs presented high expressions of CD29, CD90, and CD73, but low expressed CD34 and CD45. *P < 0.05, **P < 0.01, and ***P < 0.001

Primary SCAPs grew out from tissue after 3 days, as shown in Fig. 1a. Flow cytometry analysis showed that SCAPs positively expressed MSCs markers (CD29, CD90, and CD73), while negatively expressed the hematopoietic markers such as CD34 and CD45 (Fig. 1d). Moreover, Oil Red O, Alizarin red, and Alcian Blue staining further proved the multi-differentiation ability of SCAPs (Fig. 1c). To investigate the potential role of circSIPA1L1 in the osteogenic differentiation of SCAPs, RNA samples were collected when SCAPs were cultured in osteogenic induction medium 0, 3, and 7 days later. qRT-PCR results demonstrated that the mRNA expressions of *ALPL*, *RUNX2*, and *OSX* were increased obviously, indicating osteogenic induction of SCAPs was successful. Interestingly, the expression of circSIPA1L1 was significantly upregulated under osteogenic induction of SCAPs, while miR-204-5p exhibited the opposite trend (*P* < 0.05 or *P* < 0.01, Fig. 1b).

### CircSIPA1L1 has no effect on SCAPs proliferation

**Fig. 2:**
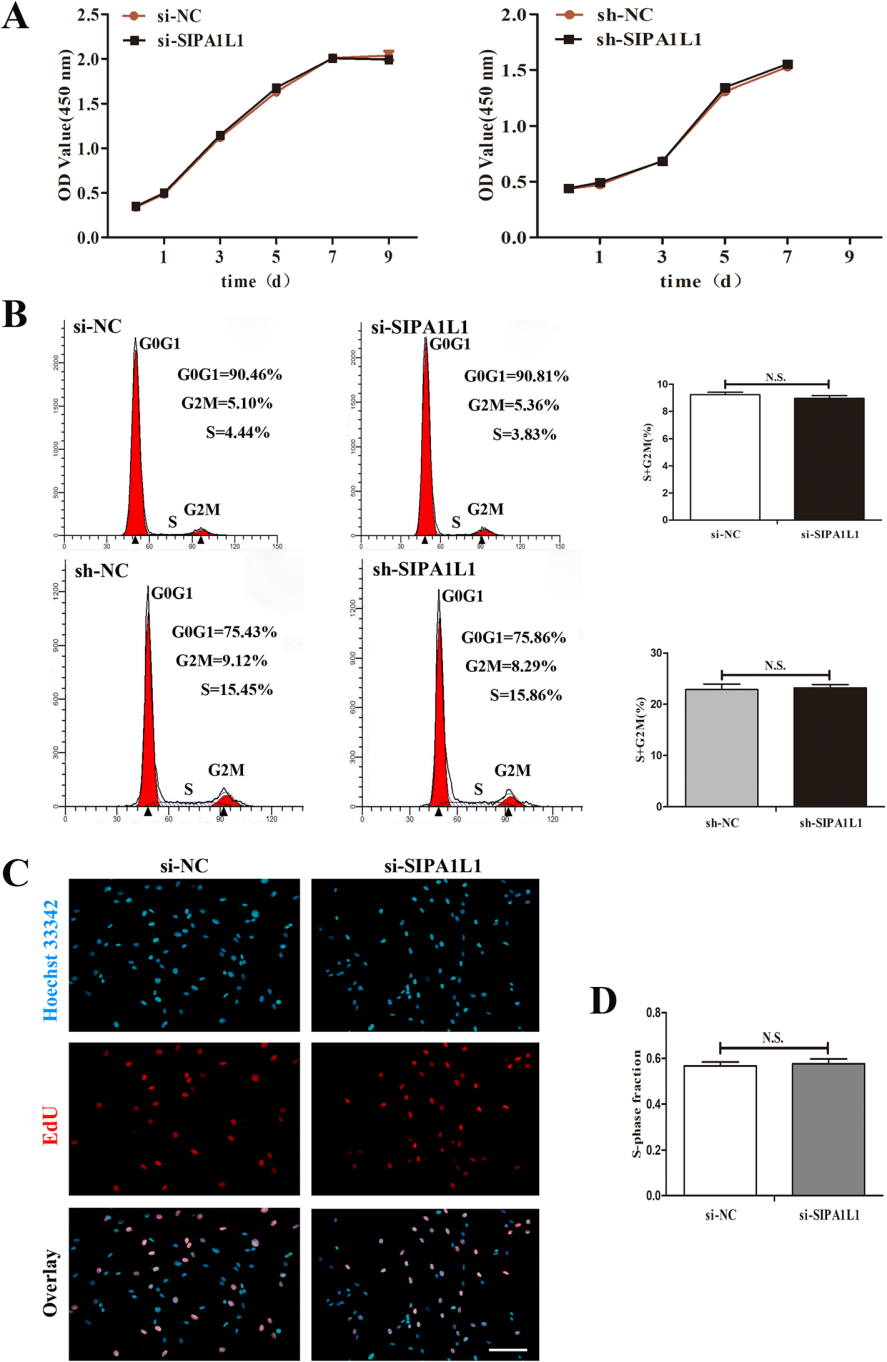
CircSIPA1L1 no effect on cell proliferation of SCAPs. **a** CCK-8 assay showed no significant difference in cell proliferation when circSIPA1L1 was overexpression or knockdown. **b**–**d** Flow cytometry and EdU assay demonstrated that circSIPA1L1 had no significant difference in cell proliferation of SCAPs. Scale Bar = 200 μm; N.S., *P* > 0.05

**Fig. 3:**
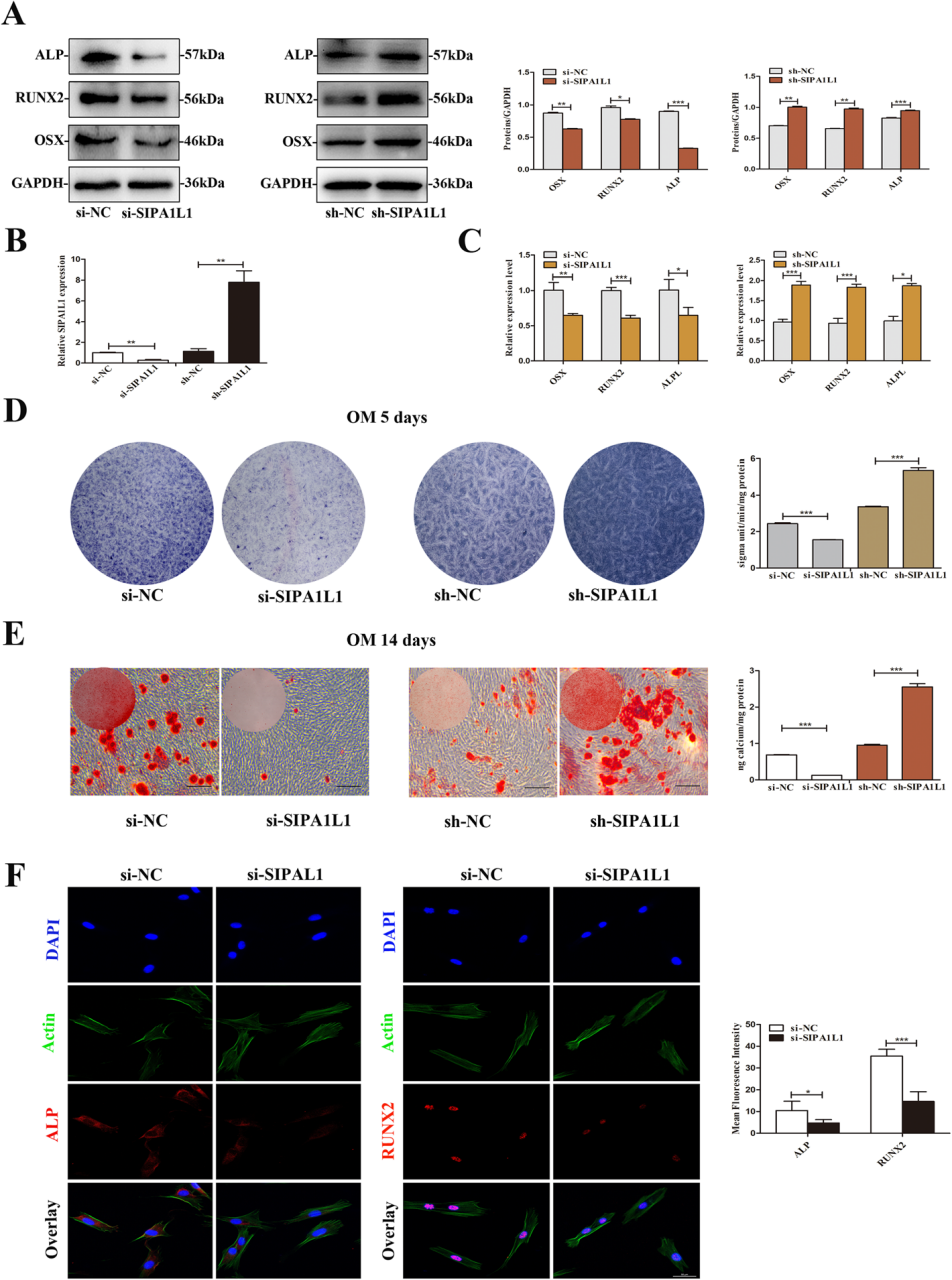
CircRNASIPA1L1 promotes osteogenic differentiation of SCAPs. **a** The protein levels of ALP, RUNX2, and OSX in the sh-SIPA1L1 group and si-SIPA1L1 group as detected by Western blotting. GAPDH served as an internal control. Histograms showed the quantification of band intensities. **b** Transfection efficacy of circSIPA1L1. **c** Relative mRNA expressions of *ALPL*, *RUNX2*, and *OSX* measured by qRT-PCR. *GAPDH* was used for normalization. **d** Images of alkaline phosphatase (ALP) staining in the different groups after being cultured in osteogenic medium (OM) for 5 days. Histograms show ALP activity by spectrophotometry. **e** Alizarin Red S staining and CPC assay were conducted to investigate the mineralization of SCAPs at day 14 (scale bar = 200 μm). **f** ALP and RUNX2 in SCAPs transfected with si-SIPA1L1 or NC were observed by immunofluorescence staining. Scale bar = 50 μm. Quantification was done by ImageJ. *P < 0.05, **P < 0.01, and ***P < 0.001

To investigate whether circSIPA1L1 can exert direct effects on the cell proliferation of SCAPs, flow cytometry assay, CCK-8 assay, and EdU retention assay were performed. SCAPs transfection was conducted to upregulate or downregulate the expression of circSIPA1L1. The transfection efficacy was obvious by qRT-PCR analysis (*P* < 0.01, Fig. 3b). As the CCK-8 assay curves showed, 100 nM circSIPA1L1 did not affect cell proliferation (Fig. 2a). In addition, EdU retention assay showed the same results (Fig. 2c, d). Flow cytometry analysis results further confirmed that no apparent difference in the proliferative index between the circSIPA1L1 group and negative control group (*P* > 0.05, Fig. 2b).

### CircSIPA1L1 promotes osteogenic differentiation of SCAPs

To assess the involvement of circSIPA1L1 in the regulation of osteogenic differentiation of SCAPs, transfection was conducted in an osteogenic medium to knockdown or overexpression the circSIPA1L1 (*P* < 0.01, Fig. 3b). Western blot results showed that expression of ALP, RUNX2, and OSX in si-SIPA1L1 was also markedly downregulated than those in the negative control group, but circSIPA1L1 overexpression obtained the opposite effects (*P* < 0.05 or *P* < 0.01, Fig. 3a). Meanwhile, the results of qRT-PCR indicated that the mRNA expressions of *ALPL*, *RUNX2*, and *OSX* were downregulated by knockdown of circSIPA1L1, whereas a significant increase was observed in overexpression of circSIPA1L1 group (*P* < 0.05 or *P* < 0.01, Fig. 3c). The results of ALP staining and ALP activity detection both presented obviously decline in the si-circSIPA1L1group compared with the si-NC group after induction for 5 days while overexpression circSIPA1L1 increased ALP activity (Fig. 3d). Besides, after 14 days of osteogenic induction, the intensity of ARS staining and matrix mineralization was distinctly decreased in the si-circSIPA1L1 group, whereas it was notably increased in circSIPA1L1 overexpression group. Moreover, CPC results also showed that the calcium concentration in circSIPA1L1 knockdown group was much lower than the control group, while it was elevated in circSIPA1L1 overexpression group (Fig. 3e). In addition, the results of the immunofluorescence assay further confirmed that the levels of ALP and RUNX2 were downregulated in the si-SIPA1L1 group (Fig. 3f). Taken together, these results demonstrated that circSIPA1L1 has a positive effect on osteogenic differentiation of SCAPs.

### Overexpression of miR-204-5p inhibits osteogenic differentiation of SCAPs

**Fig. 4:**
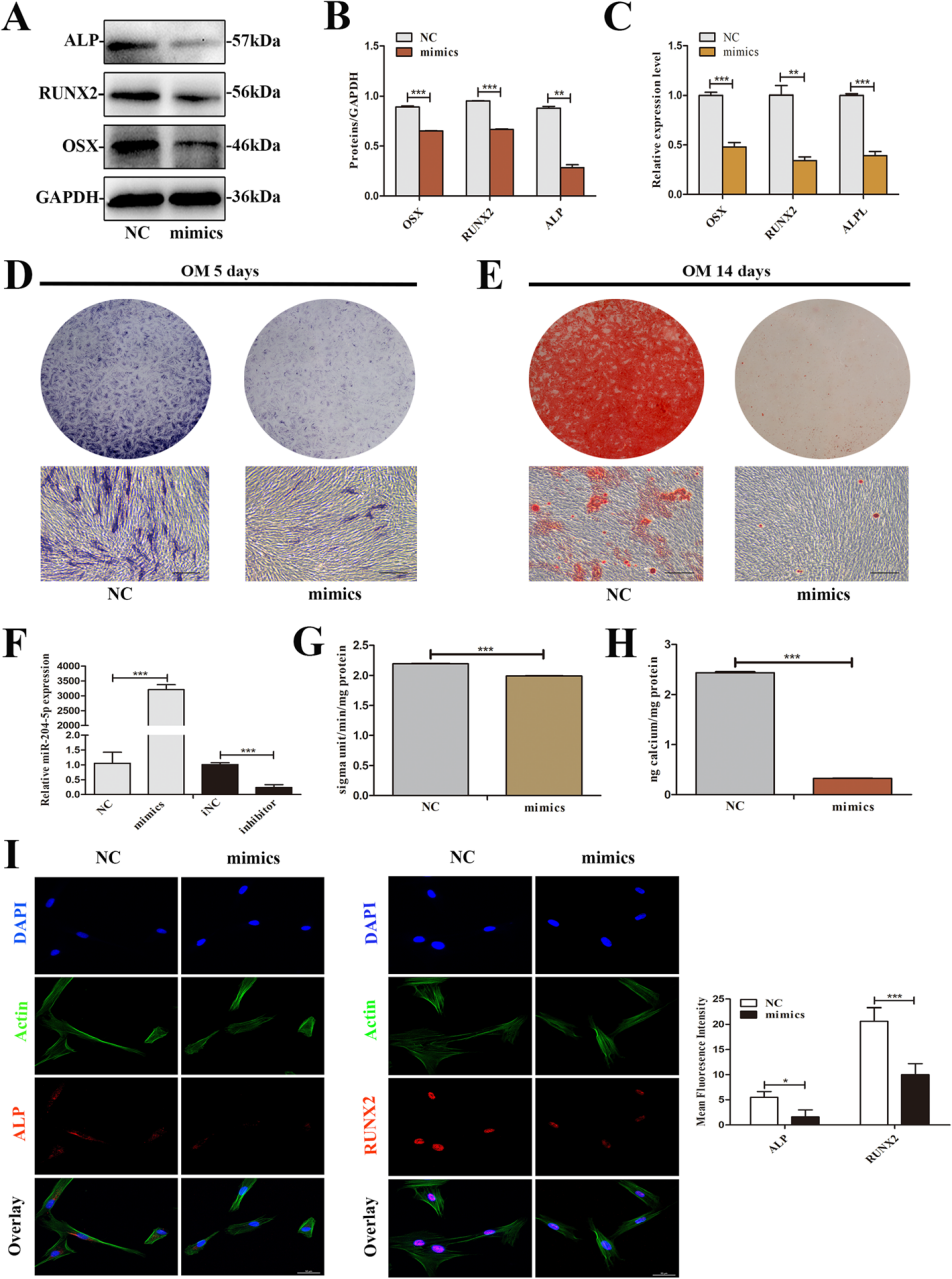
Overexpression of miR-204-5p suppresses osteogenic differentiation of SCAPs. **a** Western blot assays were performed to analyze the protein expression levels of ALP, RUNX2, and OSX in the miR-204-5p group than mimics NC group. GAPDH was the internal control. **b** Grayscale analyses. **c** qRT-PCR showed lower levels of *ALPL*, *RUNX2*, and *OSX* in the mimics group. **d**, **g** Images of ALP staining in the miR-204-5p group. SCAPs were cultured in OM for 5 days. Histograms showed ALP activity by spectrophotometry (bar = 200 μm) **e**, **h** After cell culture in OM for 14 days, Alizarin Red S staining, and CPC assay showed that miR-204-5p mimics suppresses mineralization of SCAPs (scale bar = 200 μm). **f** Transfection efficacy of miR-204-5p mimics and inhibitor. **i** Immunofluorescence staining showed negative expressions of ALP and RUNX2 in **the** miR-204-5p mimics group (scale bar = 50 μm. Quantification was done by ImageJ. *P < 0.05, **P < 0.01, ***P < 0.001

To explore the functional role of miR-204-5p in the regulation of osteoblast differentiation in SCAPs, miR-204-5p mimics was used to increased miR-204-5p expression in SCAPs. The results of qRT-PCR revealed that miR-204-5p was obviously increased in the miR-204-5p mimics group and reduced approximately 80% in the miR-204-5p inhibitor group compared with the respective NC group (*P* < 0.01, Fig. 4f). Compared with the NC group, the expression levels of osteogenic differentiation marks (ALP/*ALPL*, RUNX2/*RUNX2*, and OSX/*OSX*) were significantly decreased after additional transfection with miR-204-5p mimics (*P* < 0.01, Fig. 4a–c). In addition, miR-204-5p overexpression decreased ALP activity and reduced mineralization nodule formation compared with SCAPs transfected with mimic-NC group after 5 and 14 days, respectively (Fig. 4d, e). ALP activity assay and CPC results further confirmed these effects (*P* < 0.01, Fig. 4g, h). Similar to the results of Western blot analysis, we observed a consistent reduction in the protein levels of ALP and RUNX2 in miR-204-5p group by immunofluorescence assay (Fig. 4i).

### Knockdown of miR-204-5p promotes osteogenic differentiation of SCAPs

**Fig. 5:**
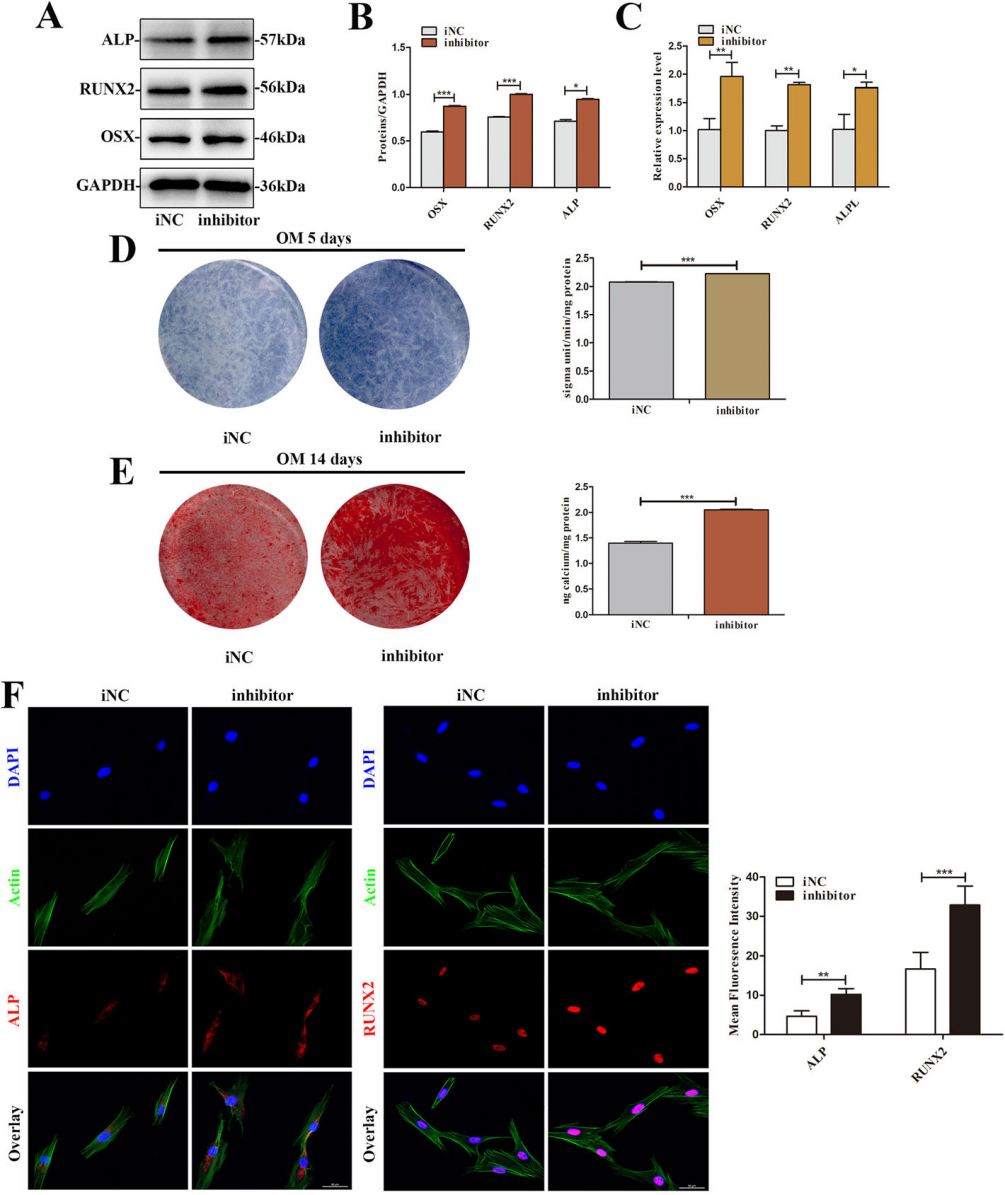
Knockdown of miR-204-5p enhanced osteogenic differentiation of SCAPs. **a** Western blot assay showed lower protein levels of ALP, RUNX2, and OSX in the inhibitor-NC group than the miR-204-5p inhibitor group. GAPDH was the internal control. **b** Grayscale analyses. **c** qRT-PCR showed higher levels of *ALPL*, *RUNX2*, and *OSX* in the miR-204-5p inhibitor group. **d** ALP staining and ALP activity assay were performed to evaluate the expression of ALP in the inhibitor-NC and miR-204-5p inhibitor group at day 5. **e** Alizarin red staining showed that the miR-204-5p inhibitor group generated more calcified nodules than the control group on day 14. CPC assay showed quantification of Alizarin red staining by spectrophotometry. **f** Immunofluorescence staining showed upregulated ALP and RUNX2 in **the** miR-204-5p inhibitor group compared with the NC group (scale bar = 50 μm). Quantification was done by ImageJ. *P < 0.05, **P < 0.01, and ***P < 0.001

SCAPs treated with miR-204-5p inhibitor and inhibitor negative control was induced to differentiate for 3 days. Both mRNA and protein levels of ALP, RUNX2, and OSX were significantly upregulated in miR-204-5p inhibitor group (*P* < 0.05 or *P* < 0.01, Fig. 5a–c). In addition, ALP staining and Alizarin red staining showed that miR-204-5p inhibitor promotes expression of ALP and formation of mineralized nodules of SCAPs (Fig. 5d, e). Immunofluorescence assay further illustrated that the protein levels of ALP and RUNX2 were upregulated in the miR-204-5p inhibitor group (Fig. 5f).

### Bioinformatic analysis of miR-204-5p target gene

**Fig. 6:**
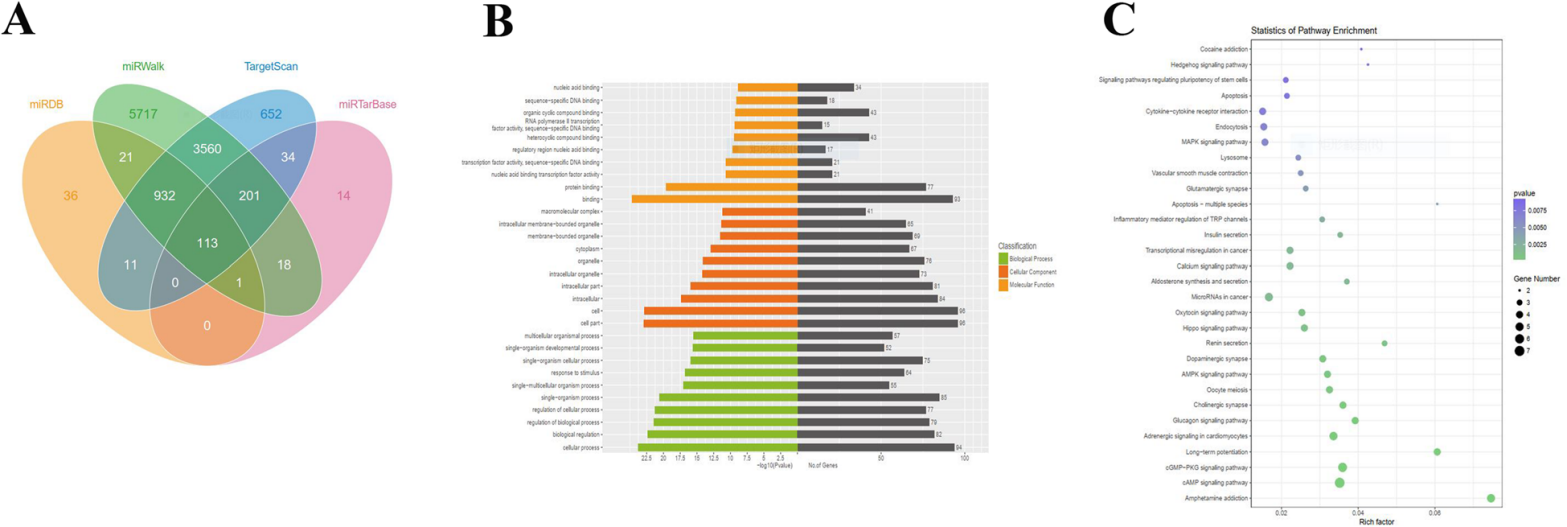
Bioinformatic analysis on the target gene of miR-204-5p. **a** Venn diagram showed the number of miR-204-5p target genes predicted by performing miRDB, miRTarBase, miRWalk, and TargetScan algorithms. **b**, **c** GO annotation (**b**) and KEGG pathway analysis (**c**) showed the top 30 target genes and their enriched pathways. GO: domains directly related with reproduction

To predict potential target genes of miR-204-5p, bioinformatics analysis was performed by TargetScan, miRWalk, miRTarBase, and MiRDB. 11,310 potential target genes were acquired, of which 113 were in common in the above 4 databases (Fig. 6a). Furthermore, GO annotation and KEGG pathway analysis showed that these target genes are involved in a multiple biological process and pathways, such as the MAPK signaling pathway that has been widely studied (Fig. 6b, c). Interestingly, ALPL as a critical gene in osteogenesis was one of the common target genes of miR-204-5p.

### CircSIPA1L1 affects the expression of ALPL by serving as a miRNA sponge for miR-204-5p

**Fig. 7:**
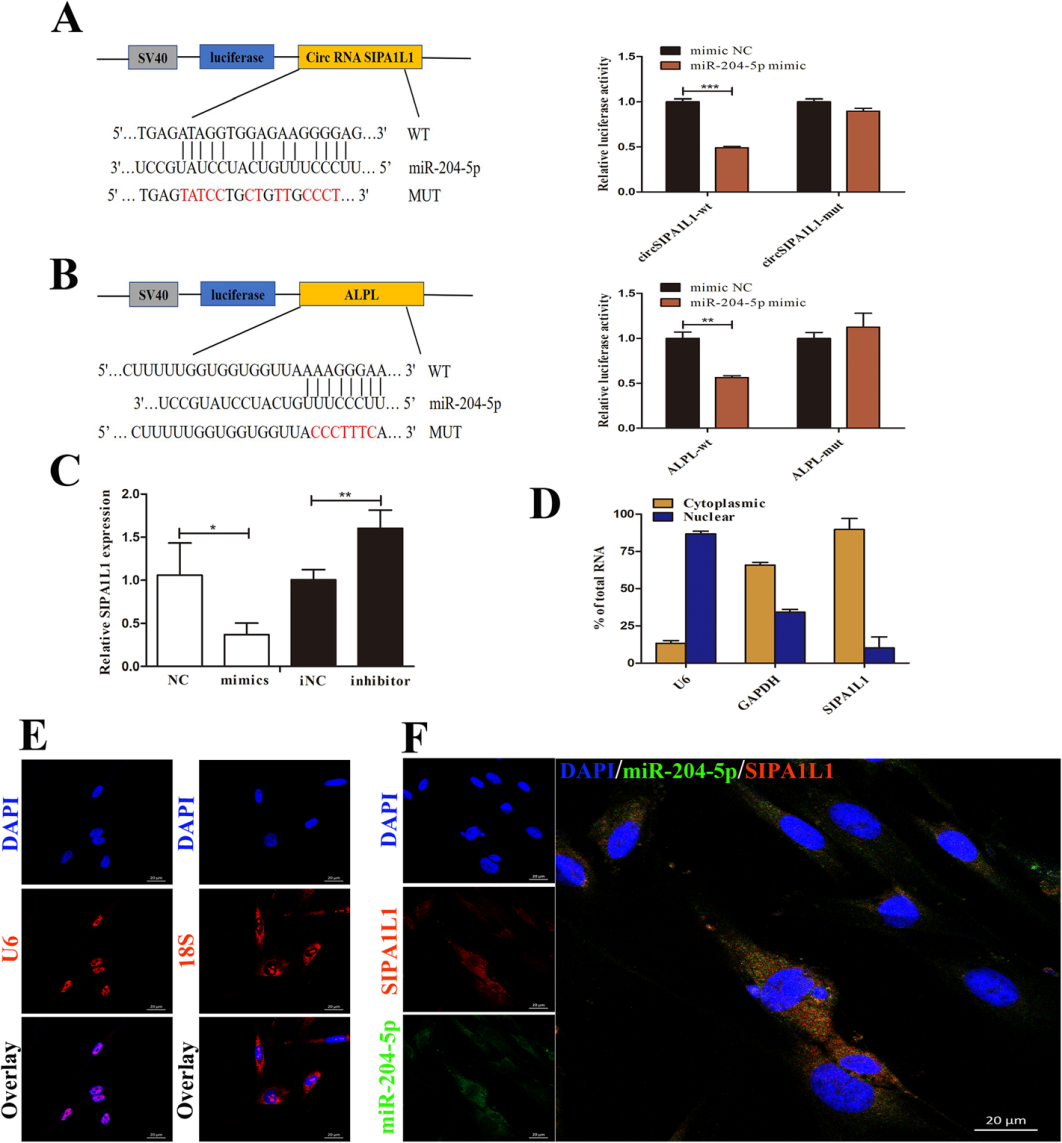
CircSIPA1L1 affects the expression of ALPL by serving as a miRNA sponge for miR-204-5p. **a** Schematic diagram of miR-204-5p with the seed sequences of wild-type circSIPA1L1 3′UTR (WT) and mutant circSIPA1L1 3′UTR (Mut). Luciferase reporter assays in 293 T illustrated that miR-204-5p could bind with circSIPA1L1. **b** The mutant ALPL-3′-UTR reporter plasmid was constructed. Luciferase reporter assays in 293 T showed that miR-204-5p could bind with ALPL. **c** Relative circSIPA1L1 expression level in SCAPs transfected with miR-204-5p mimics or inhibitor. **d** Cytoplasmic and nuclear fractions of cellular RNA were analyzed by qRT-PCR and expressed as a percentage of the input. *U6* and *GAPDH* mRNA were used as reference RNAs for nuclear and cytoplasmic fractions, respectively. **e**, **f** Co-localization between miR-204-5p and circSIPA1L1 was observed by FISH in SCAPs (**f**). 18S and U6 were the internal control (**e**). Scale bar = 50 μm. *P < 0.05, **P < 0.01

To identify the interaction of circSIPA1L1 and miR-204-5p, we simultaneously performed nuclear*/*cytoplasmic separation and FISH experiments. After separating cytoplasmic and nuclear fractions, the expression ratio of circSIPA1L1 was detected by qPCR. Almost 90% of circSIPA1L1 transcripts were measured in the cytoplasm (Fig. 7d). Furthermore, the results of FISH showed that circSIPA1L1 and miR-204-5p is mainly exist in the cytoplasm (Fig. 7e, f). These results suggested that circSIPA1L1 may have potential biological roles in post-transcriptional regulation.

To understand the underlying mechanism that circSIPA1L1 regulates osteogenic differentiation of SCAPs, we tested the expression of circSIPA1L1 after the transfection of miR-204-5p. Notably, we observed a negative correlation between circSIPA1L1 and miR-204-5p (*P* < 0.05 or *P* < 0.01, Fig. 7c). To further explore the relationship between circSIPA1L1 and miR-204-5p, the luciferase reporter plasmid was constructed by cloning the putative miR-204-5p target binding sequence of circSIPA1L1 into a luciferase plasmid vector. MiR-204-5p mimics and circSIPA1L1 wild-type or mutant luciferase plasmid were transferred into 293 T cells. MiR-204-5p mimics significantly suppressed the luciferase activity of circSIPA1L1 wild-type reporter, but this phenomenon was successfully reversed by mutation of the putative miR-204-5p target sites (*P* < 0.01, Fig. 7a). Similarly, we observed a consistent reduction of luciferase activity in 293 T co-transfected with miR-204-5p and ALPL wild-type (*P* < 0.01, Fig. 7b). Furthermore, the expression of ALPL was correspondingly negatively regulated by miR-204-5p (*P* < 0.001, Fig. 4c). Altogether, these results provided that circSIPA1L1 can affect the expression of ALPL by targeting miR-204-5p.

### CircSIPA1L1/miR-204-5p/ALPL regulates osteogenic differentiation of SCAPs

**Fig. 8:**
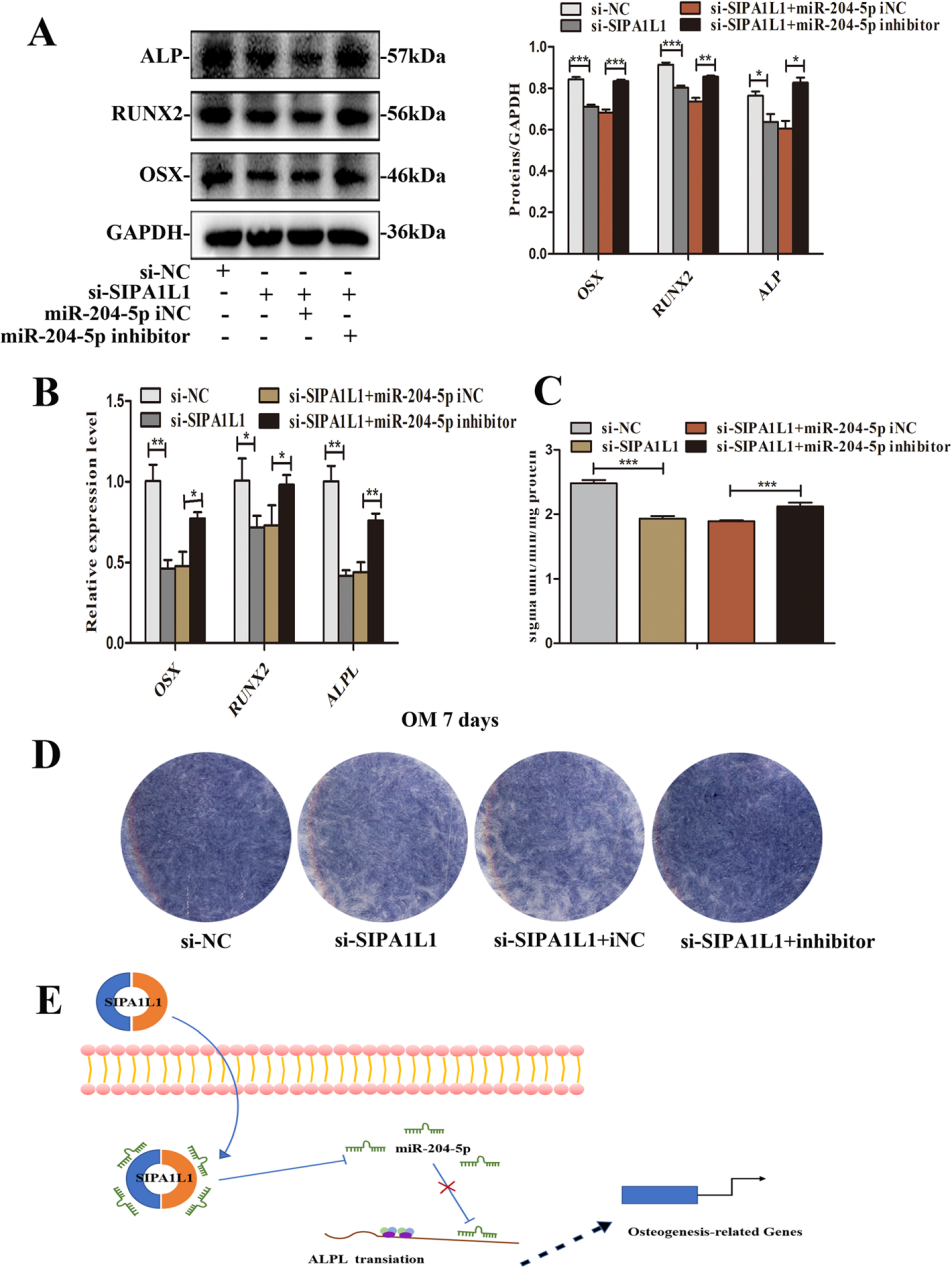
CircSIPA1L1/miR-204-5p/ALPL regulates osteogenic differentiation of SCAPs. **a** Results of western blot analysis indicated that the miR-204-5p inhibitor rescued the si-SIPA1L1 mediated downregulation of ALP, RUNX2, and OSX. GAPDH served as an internal control. Histograms showed the quantification of band intensities. **b** Results of qRT-PCR analysis revealed the miR-204-5p inhibitor rescued the si-SIPA1L1 mediated downregulation of *ALPL*, *RUNX2*, and *OSX*. **c**, **d** ALP staining and ALP activity assay showed the miR-204-5p inhibitor rescued the si-SIPA1L1 mediated downregulation expression of ALP. **e** Schematic diagram for circSIPA1L1/ miR-204-5p /ALPL axis. *P < 0.05, **P < 0.01, and ***P < 0.001

To further identify the miR-204-5p that binds to circSIPA1L1 regulating ALPL expression to promote osteoblast differentiation of SCAPs, the rescue experiments were performed. SCAPs were treated with si-SIPA1L1, miR-204-5p inhibitor, and the corresponding si-NC, inhibitor-NC. qRT-PCR and Western blot results suggested that co-transfection with si-SIPA1L1 and miR-204-5p inhibitor partially blocked the mRNA and protein expression levels of *ALPL*/ALP, *RUNX2*/RUNX2, and *OSX*/OSX, in comparison to the si-SIPA1L1 group (*P* < 0.05 or *P* < 0.01, Fig. 8a, b). As expected, ALP staining and ALP activity result further confirmed this phenomenon (Fig. 8c, d). Taken together, circSIPA1L1 absorbs miR-204-5p as a competitive endogenous RNA, increases the transcription of ALPL to promote the osteogenic differentiation of SCAPs (Fig. 8e).

## Discussion

It is well known that tissue engineering based on SCAPs has been widely reported, but the functional significance of noncoding RNA in SCAPs in tissue regeneration remains largely unknown. In our previous research, a large number of circRNAs were detected in SCAPs [31]. More importantly, we found that the expression of circSIPA1L1 was down expression while miR-204-5p was upregulated in SCAPs during the osteogenic differentiation, suggesting that circSIPA1L1 and miR-204-5p may involve in the osteogenic differentiation of SCAPs. In our study, qRT-PCR and Western blot analysis indicated that overexpression circSIPA1L1 promotes osteogenic differentiation of SCAPs in vitro. Meanwhile, ALP and ARS staining also confirmed this conclusion. Similarly, knocking down circSIPA1L1 further showed that circSIPA1L1 stimulated osteogenic differentiation. In addition, we did not observe the effect of circSIPA1L1 on cell proliferation of SCAPs. In view of the complexity of cell proliferation, whether circSIPA1L1 at different transfection concentrations or different cell lineages have different effects on proliferation may require further investigation.

We found that circSIPA1L1 and miR-204-5p were prominently co-localized in the cytoplasm of SCAPs by the FISH (Fig. 6e, f). Hence, we hypothesized that circSIPA1L1 could have functional significances in the cytoplasm. However, the function of circRNAs in the cytoplasm is diverse. Some circRNAs works in the cytoplasm to form circRNPs (circRNA-protein complexes) by adsorbing proteins [37]. For example, circFoxo3 interacted with the anti-stress proteins FAK and HIF1α, as well as the anti-senescent protein ID-1 and the transcription factor E2F1, leading to promoted cell aging [38]. In addition, circZNF609 can also actively translate to exert protein-coding ability in myoblasts, in view of the association with heavy polysomes [39]. Although almost all circular RNAs are exclusively present in the cytoplasm, there is still a small portion of circRNAs are restricted in the nucleus and bind to small nuclear ribonucleoproteins to regulate the transcription of certain genes [40].

Growing evidence identified that circRNAs are mainly located in the cytoplasm, where it affects gene expression through the action of the microRNA sponge [41]. In this research, the luciferase activity of circSIPA1L1 wild-type and miR-204-5p wild-type were remarkably decreased by miR-204-5p mimics (Fig. 6a, b). The expression of bone-related genes and proteins including *ALPL*/ALP were downregulated in the si-SIPA1L1 group, while co-transfection with miR-204-5p inhibitor and si-SIPA1L1 partially reversed this effect (Fig. 8a, b). These results indicated that circSIPA1L1 regulates ALPL expression through the classical model of sponging miR-204-5p. Interestingly, the miR-204-5p inhibitor did not completely block the effects of circSIPA1L1 knockdown, suggesting the role of circSIPA1L1 on the regulation of osteogenic differentiation could be complicated. Whether there are other regulatory mechanisms of circSIPA1L1 is worth further exploration.

According to reports, Ingenuity® Pathway Analysis results showed that miR-204-5p can directly target ALPL [28]. Here, the bioinformatics analysis and luciferase reporter experiments further proved that miR-204-5p targets the 3′UTR of the ALPL gene, inhibiting translation of ALPL at the post-transcriptional level. ALPL gene is also essential for the normal development and homeostasis of the bones and teeth, because it encodes ALP. As we all know, ALP promotes mineralization by hydrolyzing pyrophosphate to release inorganic phosphate which binds to calcium [42]. The low circulating concentration of total ALP is a characteristic feature of a rare genetic disease called hypophosphatasia that causes osteoporosis, osteomalacia, fragility fractures, and dental problems in adults [43]. The cause of hypophosphatasia is loss-of-function or dominant-negative mutations in ALPL. Heterozygous ALPL mutations can lead to low serum ALP levels. Persistent low serum ALP may be related to non-specific musculoskeletal symptoms [44]. A genetic study suggested that ALPL is necessary for postnatal bone formation. The study also further clarified that the bone deformities are related to the degree of ALPL deficiency [30]. Taken together, these results revealed that ALPL plays a critical role in bone formation. Our results show that circSIPA1L1 can significantly promote the expression of ALP, which may provide some reference for bone regeneration and even provide a new insight for the etiology and treatment of hypophosphatasia.

## Conclusion

In conclusion, our results elucidated the osteogenic function of circSIPA1L1 in SCAPs. Our findings suggest that circSIPA1L1 serves as an endogenous miR-204-5p sponge to promote osteogenic differentiation of SCAPs, which may be used as a potential target for revealing the molecular mechanism of the osteogenic differentiation of SCAPs.

## Data Availability

The datasets used and analyzed during the current study are available from the corresponding author on reasonable request.

## Abbreviations

CircSIPA1L1: Circular RNA SIPA1L1
SCAPs: Stem cells from apical papilla
ALP: Alkaline phosphatase
ARS: Alizarin red staining
MSCs: Mesenchymal stem cells
miRNA: MicroRNA
ceRNAs: Competitive endogenous RNAs
NC: Negative control
RT-PCR: Reverse transcription polymerase chain reaction
CPC: Cetylpyridinium chloride
FISH: Fluorescence in situ hybridization
